# The Impact of the COVID-19 Pandemic on Female Sexual Function in Jordan: Cross-sectional Study

**DOI:** 10.2196/40772

**Published:** 2023-02-16

**Authors:** Iman Aolymat, Lina Abdul Kadir, Mohannad Al Nsour, Hana Taha

**Affiliations:** 1 Department of Anatomy, Physiology and Biochemistry Faculty of Medicine The Hashemite University Zarqa Jordan; 2 Department of Eye and Vision Science Institute of Life Course and Medical Sciences University of Liverpool Liverpool United Kingdom; 3 Global Health Development, Eastern Mediterranean Public Health Network Amman Jordan; 4 Department of Pharmacology, Public Health and Clinical Skills, Faculty of Medicine, The Hashemite University Zarqa Jordan; 5 Department of Neurobiology, Care Sciences and Society, Karolinska Institute Stockholm Sweden; 6 Department of Family and Community Medicine, School of Medicine, The University of Jordan Amman Jordan

**Keywords:** COVID-19, pandemic, female sexual function, sexual dysfunction, stress physiology, Jordan

## Abstract

**Background:**

Sexual function is a complex physiological process controlled by neurovascular and endocrine mechanisms that are affected by stressful events. The sexual response cycle consists of four main phases, which are sexual desire or libido, arousal or excitement, orgasm, and resolution. The COVID-19 outbreak is one of the most stressful events historically, causing several unpleasant consequences, including major physical and mental disorders, and sexual dysfunction and alteration in sexual behavior are possible anticipated consequences of the pandemic. Moreover, there are social taboos related to sexual behavior in Jordan, and the current knowledge on changes in Jordanian female sexual function during COVID-19 pandemic is limited.

**Objective:**

This study aims to evaluate the impact of COVID‐19 on women's sexual function during the early COVID-19 pandemic in Jordan.

**Methods:**

This is a cross-sectional study that employed a web-based survey to follow 200 female individuals from the general population in Jordan. The survey evaluated sexual function both during COVID-19 and 6 months prior to the pandemic. The primary outcomes investigated in this study were the changes in sexual intercourse frequency and sexual function aspects, including desire, arousal, satisfaction, orgasm, lubrication, and pain during sexual activity. Data were analyzed using paired *t* test, McNemar test, Pearson correlations, and multiple linear regression using SPSS 25.

**Results:**

During the COVID-19 pandemic, the participants’ sexual intercourse frequency increased while their sexual satisfaction was significantly changed. The proportion of participants who had 0-2 times per week of sexual intercourse was decreased during the COVID-19 pandemic compared with that before the pandemic (n=90, 45% vs n=103, 51.5%; *P*=.02). Conversely, the number of female individuals with 3-7 times per week of sexual intercourse increased after the pandemic compared with the prepandemic state (n=103, 51.5% vs n=91, 45.5%; *P*=.04). Female sexual satisfaction was significantly reduced after the COVID-19 pandemic compared with that before the pandemic (3.39 vs 3.30; *P*=.049). The other categories of sexual function, including desire, arousal, satisfaction, orgasm, lubrication, and dyspareunia showed no significant changes during the COVID-19 pandemic compared with the previous 6 months. There were no significant differences between the total sexual function mean scores during COVID-19 (15.73) compared with the prepandemic scores (15.85; *P*=.41). The total score of female sexual function during the pandemic was negatively associated with the participants’ age and education level. Correlations between various demographics and sexual function categories during the COVID-19 pandemic were identified.

**Conclusions:**

This is the first study exploring female sexual function during the COVID-19 outbreak in Jordan. The results suggest that COVID-19–associated stress is influencing women's sexual function, necessitating the provision of adequate emotional and physiological well-being support for women during similar crises.

## Introduction

Sexual function is a complex physiological process that is controlled by anatomical neurological, vascular, hormonal, and psychosocial factors [1]. The sexual response cycle consists of the four main phases of sexual desire or libido, arousal or excitement, orgasm, and resolution [2]. Stress is an umbrella term that covers a broad number of factors and causes disruptions in normal physiology. The human body reacts to stressful events through the activation of the hypothalamic-pituitary-adrenal axis and the autonomic nervous system [3]. Previous research has shown that sexual function is negatively affected by stressful events; these cause fluctuations in sexual desire and psychophysiological changes to take place in the sexual response cycle [4-6].

On December 31, 2019, COVID-19 was first reported from Wuhan, China [7]. The disease is caused by a novel member of the coronavirus family called SARS-CoV-2. On March 11, 2020, the disease was confirmed as a global pandemic by the World Health Organization [7,8]. To date, the virus has infected approximately 550 million people and killed more than 6.3 million people across the world [9]. Jordan confirmed the first case of COVID-19 infection on March 2, 2020, followed by the application of strict practices related to travel, education, social events, and labor [10].

The COVID-19 outbreak is one of the most stressful events historically, causing several unpleasant consequences such as death of loved ones, major physical and mental disorders, social isolation, exceptional changes in lifestyle, job loss, and decreased income [11-14]. Therefore, sexual dysfunction and alteration in sexual behavior are possible anticipated consequences of the pandemic [15].

Public, military, and private sectors are involved in providing reproductive health services in Jordan. The continuation of sexual and reproductive health services during the COVID-19 outbreak is one of the major recommendations by the World Health Organization [16]. However, routine reproductive and sexual health services were interrupted completely or delayed during the pandemic in Jordan similar to other countries [17,18]. Deficient reproductive health services are expected to be associated with a greater risk of health problems than the SARS-CoV-2 itself.

Female individuals in Jordan represent around half of the total population, and the percentage of women of reproductive age is more than 50% of the female population in Jordan [19,20]. Worldwide, women and men are equally likely to acquire COVID-19; however, women have a lower risk of severe disease and death [7,9]. Similarly, 49% of COVID-19–confirmed cases in Jordan have been reported among female individuals [21]. Moreover, there are strong taboos against sexual behavior in the Jordanian society. Therefore, previous studies about sexual behavior, in general, are limited in Jordan, and the current knowledge on changes in the Jordanian female population’s sexual behavior during the COVID-19 outbreak is deficient. As a result, this study was designed to gain a preliminary understanding of the changes in Jordanian women’s sexual behavior in relation to the stressful COVID-19 pandemic.

This study examines the changes in sexual intercourse frequency, desire, arousal, satisfaction, orgasm, lubrication, and dyspareunia both 6 months before and after the pandemic. It also evaluates the association between the reported changes in sexual behavior and the sociodemographic characteristics of Jordanian female individuals. This research will provide the relevant baseline data regarding the impact of the pandemic on sexual health in Jordan, which will enable Jordanian authorities to provide better future support for women’s emotional and physiological well-being during crisis times.

## Methods

### Study Design and Population

This study employed a cross-sectional design, using a web-based survey to follow 200 married female individuals from the general Jordanian population. The inclusion criteria were married women at reproductive age, aged 18 years and older. The exclusion criteria were those who were younger that 18 years old and postmenopausal women. The questionnaire was developed in English, based on the literature [22,23], and then translated into Arabic by a professional translator. The questionnaire was pilot-tested randomly on 30 women. Based on the feedback, the questionnaire was adjusted and validated. Responses from the pilot study were excluded from the final analysis. The reliability (internal consistency) of the questions was estimated using Cronbach alpha (α). The Cronbach α reliability coefficient for the questionnaire was found to be ≥.82.

To avoid close contact with other people and the spread of COVID-19 through papers, and due to the lockdown and COVID-19–related restrictions, Google Forms were used to build the web-based survey.

The questionnaire included questions about the sociodemographic features of the study participants, as well as questions with short answer or multiple-choice questions about the participant’s menstruation, genital tract health, contraception use (which was published elsewhere [24]), pregnancy and childbirth, reproductive attitudes, and sexual function. The sexual function assessment evaluates sexual intercourse frequency and other physiological aspects related to sexual function. As such, the sexual function assessment covered sexual intercourse frequency and the following 6 items of female sexual functioning: desire, arousal, satisfaction, orgasm, lubrication, and pain during sexual activity.

### Data Collection and Processing

The data for this study were collected in September 2020, in Jordan. Social media platforms were used to invite women from the general population in Jordan to participate in the web-based questionnaire. Married female individuals who were aged ≥18 years and not menopausal were selected to take part in this study.

### Statistical Analysis

SPSS version 25 (IBM Corporation) was used to analyze the data. Descriptive analysis was conducted for sociodemographic, medical history, sexual intercourse frequency, and sexual function. A scoring system was used to assess female sexual function. The answers to desire, arousal, satisfaction, and orgasm questions were assigned from 1 to 5 points, whereas the answers to lubrication and pain questions were assigned from 0 to 5 points [22]. Higher female sexual function scores indicate better female sexual function. Data were expressed as frequency (n) and percentage (%) or mean (SD). The statistical tests of McNemar and paired sample *t* test (1-tailed) were used to analyze the data to identify the impact of the COVID-19 pandemic on sexual function. Pearson correlation coefficient and multiple linear regression were employed to explore the association between sociodemographic and female sexual function during COVID-19. No missing data were reported. A cutoff value of *P*≤.05 was regarded as statistically significant.

### Ethics Approval

This research has been reviewed and approved by the Hashemite University Institutional Review Board Committee (IRB approval number: 6/15/2019/2020). The first part of the web-based questionnaire included an introduction about the study objectives, confidentiality of data, anonymity of participation, voluntary involvement in the survey, and full autonomy to withdraw from the study at any time. Prior to progression to the other questionnaire sections, an informed electronic written consent was obtained from all the participants.

## Results

### Demographic Characteristics of Study Participants

A total of 200 female individuals aged ≥18 years from the general population participated in this study. The age distribution showed that 2.5% (n=5) of the respondents were aged 18-24 years, 70% (n=140) were aged 25-34 years, 19.5% (n=39) were aged 35-44 years, and 8% (n=16) were aged 45 years and older. Most of the participants (n=140, 70%) who participated in this study have completed their undergraduate studies. Moreover, 154 (77%) participants were living in urban areas. Other demographic features of the study participants are presented in Table 1.

**Table 1:** Demographic characteristics of the study participants (N=200).

Characteristics			Values
**Age (years) groups, n (%)**			
	18-24	5 (2.5)	
	25-34	140 (70)	
	35-44	39 (19.5)	
	≥45	16 (8)	
**Education level, n (%)**			
	Secondary or less	9 (4.5)	
	Undergraduate	140 (70)	
	Postgraduate	51 (25.5)	
**Place of residence, n (%)**			
	Urban	154 (77)	
	Rural	46 (23)	
Age (years) at first menstruation, mean (SD)			13.48 (1.8)
Age (years) at marriage, mean (SD)			24 (3.1)
Gravidity, mean (SD)			2.9 (1.9)
Number of abortions, mean (SD)			0.5 (0.8)
Number of vaginal births, mean (SD)			1.7 (1.5)
Number of caesarean deliveries, mean (SD)			0.7 (1.0)
**Chronic diseases, n (%)**			
	No	187 (93.5)	
	Yes	13 (6.5)	

### COVID-19 and Sexual Function

Table 2 summarizes the female sexual function. Regarding the sexual intercourse frequency, the proportion of participants who had 0-2 times per week of sexual intercourse was decreased during the COVID-19 pandemic compared with before the pandemic (n=90, 45% vs n=103, 51.5%; *P*=.02). Conversely, the number of participants with 3-7 times per week of sexual intercourse was increased after the pandemic in comparison to the prepandemic state (n=103, 51.5% vs n=91, 45.5%; *P*=.04). The proportion of participants who had sexual intercourse frequency of more than 7 times per week was not significantly changed during the pandemic in comparison to before the pandemic. The total sexual function mean scores during the COVID-19 pandemic (15.73) were nearly similar to the prepandemic scores (15.85; *P*=.41). Female sexual satisfaction was significantly reduced after the COVID-19 pandemic compared with before the pandemic (3.39 vs 3.30; *P*=.049). Other categories of sexual function, including desire, arousal, satisfaction, orgasm, lubrication, and dyspareunia showed no significant changes during the COVID-19 pandemic in comparison to 6 months before.

**Table 2:** Comparison of the participant’s sexual function before and after the COVID-19 pandemic (N=200).

Variables			Six months before COVID-19 (control)		Six months after COVID-19		*P* value
**Sexual intercourse frequency/week, n (%)**							
	0-2 times	103 (51.5)		90 (45)		.02	
	3-7 times	91 (45.5)		103 (51.5)		.04	
	>7 times	6 (3)		7 (3.5)		>.99	
Desire, mean (SD)			3.04 (0.766)		3.07 (0.747)		.37
Arousal, mean (SD)			3.11 (0.786)		3.05 (0.731)		.22
Orgasm, mean (SD)			3.08 (0.835)		3.11 (0.819)		.24
Satisfaction, mean (SD)			3.39 (0.902)		3.30 (0.897)		.049
Lubrication, mean (SD)			1.56 (1.124)		1.57 (1.123)		.76
Pain, mean (SD)			3.97 (1.401)		4.11 (1.302)		.06
Total sexual function score, mean (SD)			15.85 (3.129)		15.73 (3.104)		.41

### Association Between the Sociodemographic Characteristics of Study Participants and Sexual Function During COVID-19

The bivariate associations using Pearson correlation coefficient between the sociodemographic variables and female sexual function during the COVID-19 pandemic are displayed in Table 3. The bivariate associations suggested a positive correlation between the sexual intercourse frequency during the pandemic and the age at menarche. A positive correlation between sexual desire during the pandemic and the location of residency was also identified. On the other hand, sexual orgasm during COVID-19 and marriage age were negatively associated. Female sexual satisfaction during COVID-19 was inversely associated with the participants’ age and marital age, while lubrication during COVID-19 was negatively linked with the level of education and place of residency. Further positive correlations were observed between dyspareunia during the COVID-19 pandemic and each of the following variables: participants’ age, level of education, gravidity, number of vaginal births, and chronic diseases. Finally, total scores of female sexual function during the pandemic were negatively associated with participants’ age and education level.

**Table 3:** Association between sociodemographic features of the study participants and sexual function during COVID-19 using Pearson correlations.

	Sexual intercourse frequency/week (*r*)	Sexual desire (*r*)	Sexual arousal (*r*)	Orgasm (*r*)	Satisfaction (*r*)	Lubrication (*r*)	Pain (*r*)	Total sexual function score (*r*)
Age	–0.240	–0.109	–0.090	–0.077	–0.220^a^	–0.079	0.139^b^	–0.218^a^
Education level	–0.081	–0.052	–0.018	–0.056	–0.062	–0.158^b^	0.216^a^	–0.161^b^
Place of residence	0.066	0.188^a^	0.138	0.072	0.135	–0.193^a^	0.063	–0.005
Age at menarche	0.146^b^	0.012	–0.016	–0.093	–0.033	–0.050	–0.007	–0.070
Age at marriage	–0.101	–0.122	–0.117	–0.143^b^	–0.185^a^	–0.011	–0.017	–0.138
Gravidity	–0.089	–0.049	–0.023	0.019	–0.090	–0.001	0.142^b^	–0.079
No. of abortions	–0.092	–0.060	–0.040	–0.033	–0.090	–0.065	0.109	–0.120
No. of vaginal births	–0.046	–0.041	–0.005	0.051	–0.059	0.033	0.162^b^	–0.023
No. of caesarean deliveries	–0.054	0.033	0.006	–0.020	–0.017	–0.076	–0.074	–0.067
Chronic diseases	–0.058	0.002	0.008	–0.011	–0.088	–0.080	0.149^b^	–0.094

^a^Correlation is significant at the .01 level.

^b^Correlation is significant at the 0.05 level.

Tables 4-6 represent the multiple linear regression model between the sociodemographic characteristics of the study participants and female sexual function during the COVID-19 pandemic. The multiple linear regression model revealed that with older age, participants were more likely to have lower sexual intercourse frequencies during the COVID-19 pandemic (B=–0.249, 95% CI –0.405 to –0.093, *P*=.002), whereas participants with older ages at menarche were more likely to have higher sexual intercourse frequencies during the pandemic (B=0.055, 95% CI 0.011 to 0.099, *P*=.01). Those who were located in urban areas were more likely to have lower sexual desire during the pandemic (B=0.323, 95% CI 0.071 to 0.575, *P*=.01). The results have also indicated that, during the COVID-19 pandemic, the need for lubrication during sexual intercourse was higher in those with lower education level (B=–0.373, 95% CI –0.700 to –0.046, *P*=.03) and living at urban areas (B=–0.524, 95% CI –0.898 to –0.149, *P*=.006). Finally, the multiple linear regression model also reported that those with higher education levels were more likely to develop dyspareunia during the COVID-19 pandemic.

**Table 4:** Association between sociodemographic features of the study participants and sexual function during COVID-19 using multiple linear regression.

Variable	Frequency					Desire					Arousal			
	B	SE	*P* value	95% CI	B		SE	*P* value	95% CI	B		SE	*P* value	95% CI
Age	–0.249	0.079	.002	–0.405 to –0.093	–0.123		0.107	.25	–0.335 to 0.088	–0.106		0.106	.32	–0.316 to 0.103
Education level	–0.032	0.082	.70	–0.194 to 0.131	–0.0000685		0.112	>.99	–0.220 to 0.220	0.047		0.110	.67	–0.171 to 0.265
Place of residence	0.057	0.094	.54	–0.128 to 0.243	0.323		0.128	.01	0.071 to 0.575	0.240		0.126	.06	–0.009 to 0.490
Age at menarche	0.055	0.022	.01	0.011 to 0.099	–0.005		0.030	.88	–0.064 to 0.055	–0.015		0.030	.61	–0.074 to 0.044
Age at marriage	0.001	0.015	.96	–0.029 to 0.030	–0.024		0.020	.24	–0.064 to 0.016	–0.025		0.020	.21	–0.065 to 0.014
Gravidity	0.008	0.043	.85	–0.076 to 0.093	–0.019		0.059	.74	–0.135 to 0.096	–0.013		0.058	.82	–0.127 to 0.101
No. of abortions	–0.038	0.066	.57	–0.168 to 0.093	–0.008		0.090	.93	–0.186 to 0.169	–0.004		0.089	.97	–0.179 to 0.172
No. vaginal births	0.015	0.055	.79	–0.093 to 0.123	0.014		0.074	.85	–0.133 to 0.161	0.019		0.074	.80	–0.127 to 0.164
No. of caesarean deliveries	0.003	0.060	.95	–0.114 to 0.121	0.043		0.081	.60	–0.118 to 0.203	0.025		0.080	.75	–0.133 to 0.184
Chronic diseases	0.080	0.169	.64	–0.252 to 0.413	0.199		0.229	.39	–0.253 to 0.651	0.184		0.227	.42	–0.263 to 0.631

**Table 5:** Association between sociodemographic features of the study participants and sexual function during COVID-19 using multiple linear regression.

Variable	Orgasm					Satisfaction					Lubrication			
	B	SE	*P* value	95% CI	B		SE	*P* value	95% CI	B		SE	*P* value	95% CI
Age	–0.033	0.121	.79	–0.272 to 0.207	–0.243		0.127	.06	–0.494 to 0.007	–0.095		0.159	.55	–0.410 to 0.219
Education level	–0.091	0.126	.47	–0.340 to 0.157	0.044		0.132	.74	–0.216 to 0.305	–0.373		0.166	.03	–0.700 to –0.046
Place of residence	0.007	0.144	.96	–0.278 to 0.291	0.281		0.151	.06	–0.017 to 0.580	–0.524		0.190	.006	–0.898 to –0.149
Age at menarche	–0.042	0.034	.22	–0.109 to 0.026	–0.020		0.036	.57	–0.091 to 0.050	–0.006		0.045	.90	–0.094 to 0.083
Age at marriage	–0.037	0.023	.11	–0.083 to 0.008	–0.043		0.024	.08	–0.090 to 0.004	0.028		0.030	.35	–0.031 to 0.088
Gravidity	–0.052	0.066	.43	–0.182 to 0.079	–0.012		0.069	.86	–0.148 to 0.125	0.065		0.087	.46	–0.107 to 0.236
No. of abortions	0.017	0.102	.87	–0.183 to 0.217	–0.033		0.106	.76	–0.243 to 0.177	–0.153		0.134	.26	–0.416 to 0.111
No. of vaginal births	0.056	0.084	.50	–0.109 to 0.222	0.002		0.088	.98	–0.172 to 0.175	0.004		0.110	.97	–0.214 to 0.222
No. of caesarean deliveries	0.019	0.092	.84	–0.162 to 0.200	0.017		0.096	.86	–0.172 to 0.207	–0.070		0.121	.56	–0.308 to 0.168
Chronic diseases	–0.003	0.259	.99	–0.513 to 0.508	0.028		0.271	.92	–0.507 to 0.563	–0.297		0.340	.38	–0.968 to 0.374

**Table 6:** Association between sociodemographic features of the study participants and sexual function during COVID-19 using multiple linear regression.

Variable	Pain				Total sexual function score			
	B	SE	*P* value	95% CI	B	SE	*P* value	95% CI
Age	0.121	0.183	.51	–0.240 to 0.481	–0.843	0.443	.06	–1.715 to 0.030
Education level	0.584	0.190	.002	0.209 to 0.959	–0.664	0.460	.15	–1.572 to 0.244
Place of residence	0.260	0.218	.23	–0.169 to 0.690	–0.035	0.527	.95	–1.075 to 1.004
Age at menarche	–0.035	0.051	.50	–0.136 to 0.067	–0.091	0.124	.47	–0.337 to 0.154
Age at marriage	–0.043	0.035	.21	–0.111 to 0.025	–0.049	0.084	.56	–0.214 to 0.116
Gravidity	0.050	0.100	.62	–0.147 to 0.246	0.045	0.241	.85	–0.431 to 0.521
No. of abortions	0.061	0.153	.69	–0.241 to 0.363	–0.357	0.371	.34	–1.088 to 0.374
No. of vaginal births	0.001	0.127	.99	–0.249 to 0.251	0.058	0.307	.85	–0.547 to 0.663
No. of caesarean deliveries	–0.133	0.138	.34	–0.406 to 0.140	–0.073	0.335	.83	–0.733 to 0.588
Chronic diseases	0.663	0.390	.09	–0.107 to 1.432	–0.102	0.944	.91	–1.964 to 1.761

## Discussion

### Principal Findings

Using a cross-sectional survey, this work explored the impact of the COVID-19 pandemic–related stressors on female sexual function in Jordan. Generally, the early COVID-19 pandemic era was associated with changed patterns of sexual intercourse frequency with a relative shift toward having intercourse 3-7 times per week. The COVID-19 outbreak was also associated with lower female sexual satisfaction. Our study explored whether the sociodemographic factors of the study participants could affect female sexual function during the pandemic.

In this study, it was observed that female sexual intercourse frequency pattern was shifted from 0-2 times per week before the pandemic to 3-7 per week during the pandemic, which suggests increased frequency of sexual intercourse during COVID-19. This change in sexual intercourse frequency is probably due to the lockdowns and social isolation, where more time is spent with partners at home during the pandemic. Nevertheless, contrasting results in the scientific literature about the impact of the COVID-19 pandemic on the frequency of sexual intercourse are present. Similarly, other studies carried out in Singapore [23], Turkey [22], Bangladesh, India, and Nepal [25] reported that weekly sexual frequency was increased during the COVID-19 pandemic. However, other studies conducted in China [26], the United States, Canada, Australia, and the United Kingdom [27] revealed decreased sexual intercourse frequency during the COVID-19 pandemic. The conflicting results regarding changes in sexual frequency could perhaps be explained by the cultural and religious perceptions around sexual activity.

Unpleasant way of life is known to influence female sexual function [28]. However, contrasting results in previous research have been identified. For example, a study reported that Gulf War veterans with chronic fatigue experienced low libido and abnormal vaginal lubrication but no significant change in their satisfaction [29]. Another study reported that fatigue and stress were found to have a significant association with dyspareunia [30]. Additionally, infertility-related stress significantly reduced female sexual desire, arousal, orgasm, and sexual satisfaction, but increased the incidence of pain during intercourse and the need for lubrication [31]. Unemployment, trauma exposure, and chronic stress were also associated with low sexual desire, satisfaction, and arousal [5,31-33]. The findings of this study illustrate that female individuals experienced decreased levels of sexual satisfaction during the COVID-19 pandemic. The results of this study agree with those of previous studies conducted in Turkey [22], Italy [34], and Taiwan [35] during the COVID-19 outbreak. Moreover, this research revealed that the scores of other categories of sexual function, including desire, orgasm, pain, and lubrication, were not changed during the COVID-19 pandemic. By contrast, Yuksel et al [22] reported that the COVID-19 pandemic was associated with a significant reduction in female desire, arousal, and orgasm scores, but no change in lubrication and pain scores during the pandemic were shown. On the other hand, Ballester-Arnal et al [36] stated that no change in female sexual desire, arousal, and pain scores were identified, but a reduction in lubrication, orgasm, and satisfaction scores during the pandemic were observed. Furthermore, in this study, the total female sexual function scores were not changed during the pandemic compared with before that. On the other hand, previous reports in the literature revealed that the total female sexual function scores during the pandemic were significantly decreased [22,34,36]. The variation in these results could be explained by the variation in the study design, demographic features, religious coping, and the extent and timing of the pandemic restrictions.

This study revealed that some sociodemographic features of our study participants appeared to be significantly related to their sexual function. Several studies have already investigated the impact of sociodemographic factors on sexual function. However, the findings of these various studies are variable and inconsistent. Ter Kuile et al [37] showed that the female sexual function categories, including desire, arousal, lubrication, orgasm, and satisfaction, were negatively correlated with age. Arasteh et al [38] also reported that age was negatively correlated with desire, arousal, lubrication, orgasm, and satisfaction. They also showed that gravidity was negatively correlated with desire, arousal, and satisfaction. They also stated that age at marriage was positively correlated with arousal and orgasm, while it was negatively linked with satisfaction and pain. Furthermore, their study has revealed that each parameter of desire, arousal, and orgasm was proportionally related with the level of education, whereas both pain and satisfaction were negatively linked with education level [38]. Nevertheless, other studies stated that sexual satisfaction was not affected by age, level of education, or marital status [39], and that age at menarche showed no impact on coital frequency [40]. Moreover, studies in the past have found that the location of residency had an impact on sexual function [41] and that sexual function was reduced in women with older ages and those having medical and psychological disorders [42]; sexual function was also shown to be increased in those with older ages at marriage [38]. Only a few studies have evaluated female sexual function during the stressful COVID-19 outbreak [22,43], and little is known about the impact of the sociodemographic factors on female sexual function during the COVID-19 pandemic.

It is a possibility that women who are not satisfied with their sexual behavior and function could have unrelated personal and interpersonal discomforts. In addition to mental health disorders, social problems are another major consequence that could result from sexual dysfunction [44]. Social problems could include divorce and family disintegration, forced sex, and sex outside of marriage, which is extremely forbidden in Jordan and could instigate honor crimes [45]. Therefore, understanding the variables that affect women’s sexual function could help in providing women with the appropriate measures such as psychological support, counseling, education, and training during critical situations. This will improve women’s sexual function and reduce the negative impacts on mental health and social integrity.

### Limitations

Despite this being the first study exploring female sexual function during the COVID-19 outbreak in Jordan, our findings have potential limitations. For example, the sample size is small and not representative; therefore, the results cannot be generalized to the whole population. This limitation can be explained by the fact that the Jordanian society has conservative views around sexuality and that the Jordanian population frown upon discussing their sexual behavior with strangers [45]. As a result, when participants started the survey and reached the sexual function section, there was a big possibility that they left the survey before completing it. Another limitation could be that the questionnaire was completed without direct contact with the participants. This could have contributed to self-reporting bias and misunderstanding of some of the questions and therefore inappropriate responses. However, due to the sensitivity of the research topic in the Jordanian context, self-reporting gave the respondents the freedom of answering the questions without observation by anyone from the research team and this might have reduced the social desirability bias. Finally, there might have been a recall bias by the participants related to their responses to the prepandemic questions of the survey.

### Conclusions

In conclusion, this study showed that early COVID-19 prevalence in Jordan was associated with a change in sexual frequency patterns. Sexual satisfaction of Jordanian women was also compromised during the pandemic. Furthermore, sociodemographic factors seem to have an impact on the sexual function of women during the pandemic. These results can guide the Jordanian authorities to provide future support together with sexual and reproductive health services for women in pandemic situations. We also recommend conducting further research to assess the impact of the COVID-19 pandemic on female sexual function given the different time intervals beyond the first 6 months of the pandemic.
